# Long-Term Pulmonary Function after Combined Anteroposterior Spinal Fusion for Scoliosis in Neurofibromatosis Type 1 and Marfan Syndrome at a Mean Follow-Up of 13 Years

**DOI:** 10.1055/s-0045-1809340

**Published:** 2025-07-06

**Authors:** José Alberto Alves Oliveira, Rogério dos Reis Visconti, Gustavo Borges Laurindo de Azevedo, Alderico Girão Campos de Barros, Luis E. Carelli, José Roberto Lapa e Silva

**Affiliations:** 1 School of Medicine, Universidade Federal do Rio de Janeiro, Rio de Janeiro, RJ, Brazil; 2 Instituto Nacional de Traumatologia e Ortopedia Jamil Haddad, Rio de Janeiro, RJ, Brazil

**Keywords:** Marfan syndrome, neurofibromatosis type 1, pulmonary function, scoliosis, spinal fusion, escoliose, função pulmonar, fusão espinal, neurofibromatose tipo 1, síndrome de Marfan

## Abstract

**Objective:**

To compare the effect of combined spinal fusion (anteroposterior) on pulmonary function in patients with scoliosis secondary to Marfan syndrome versus neurofibromatosis type 1 (NF1) at long-term follow-up (> 10 years).

**Methods:**

Retrospective comparative study with nine patients, operated from March 1997 to December 2009, groups: Marfan syndrome versus NF1. Outcome measures were sex; age (at diagnosis and surgery); corrected height by wingspan; body mass index (BMI); duration of surgery (minutes); estimated blood loss (mL); last follow-up (years); pulmonary and implants related complications; pre- and postoperative Cobb angle of main thoracic curve and of thoracic kyphosis (T5 to T12); number of instrumented levels; absolute and percentage predicted values of forced vital capacity (FVC) and forced expiratory volume in 1 second (FEV1). The data was processed in the IBM SPSS Statistics for Windows software (IBM Corp.), version 20.0, and the comparisons of means used the Student's *t* -test and analysis of variance (ANOVA), or the Mann-Whitney and Kruskal-Wallis/Dunn tests, with a *p* -value of 0.05.

**Results:**

There was no difference in the absolute and predicted percentage values of pulmonary function, FVC and FEV1, and Cobb of the main thoracic curve between the groups, pre- and postoperatively ( *p* > 0.05). However, there was a significant reduction in the Cobb of the main thoracic curve in the Marfan syndrome group (74→46°, *p* < 0.05).

**Conclusion:**

There was no worsening of pulmonary function in patients who underwent the combined approach after a follow-up of more than 10 years, and there were no significant differences in the postoperative values of the pulmonary function test between the groups.

Level of evidence IV; case series.

## Resumo

**Objetivo:**

Comparar o efeito da fusão espinhal combinada (anteroposterior) na função pulmonar em pacientes com escoliose secundária à síndrome de Marfan ou à neurofibromatose tipo 1 (NF1) no acompanhamento em longo prazo (> 10 anos).

**Métodos:**

Estudo retrospectivo comparativo com nove pacientes, operados entre março de 1997 a dezembro de 2009. Os pacientes foram divididos em grupos com síndrome de Marfan ou NF1. As medidas de desfecho incluíram sexo, idade (ao diagnóstico e à cirurgia), altura corrigida pela envergadura, índice de massa corporal (IMC), duração da cirurgia (minutos), perda sanguínea estimada (mL), último acompanhamento (anos), complicações pulmonares e relacionadas aos implantes, ângulo de Cobb pré e pós-operatório da curva torácica principal e da cifose torácica (T5 a T12), número de níveis instrumentados, valores absolutos e percentuais previstos de capacidade vital forçada ( *forced vital capacity* , FVC, em inglês) e volume expiratório forçado em um segundo ( *forced expiratory volume in 1 second* , FEV1, em inglês). Os dados foram processados no programa IBM SPSS Statistics for Windows (IBM Corp.), versão 20.0, e as comparações de médias empregaram o teste *t* de Student e a análise de variância ( *analysis of variance* , ANOVA, em inglês), ou os testes de Mann-Whitney e Kruskal-Wallis/Dunn com valor de *p* de 0,05.

**Resultados:**

Não houve diferença nos valores percentuais e absolutos previstos da função pulmonar, ou seja, FVC e FEV1, e no ângulo de Cobb da curva torácica principal entre os grupos nos períodos pré e pós-operatório ( *p* > 0,05). Entretanto, houve redução significativa do ângulo de Cobb da curva torácica principal no grupo com síndrome de Marfan (74→46°; *p* < 0,05).

**Conclusão:**

Não houve piora da função pulmonar nos pacientes submetidos à abordagem combinada após um acompanhamento de mais de 10 anos. Além disso, não houve diferenças significativas nos valores pós-operatórios dos testes de função pulmonar entre os grupos.

Nível de evidência IV; série de casos.

## Introduction

Scoliosis affects about 62% of patients with Marfan syndrome, and is the most common spinal deformity. 1 2 In patients with moderate or severe deformity of the rib cage, whether due to scoliosis, pectus excavatum, or both, is common to have a restrictive disorder of pulmonary function. 3 4

Whereas in neurofibromatosis type I (NF1), Von Recklinghausen's disease, the prevalence of scoliosis is 10 to 60%, and it can be nondystrophic or dystrophic. In these cases, there may be a significant impairment of the pulmonary function of these patients if three or more dysplastic features are present—short, sharp, single thoracic curve; rib penciling; wedging of one or more vertebral bodies; masses in paraspinal or intraspinal soft tissues; marked rotation at the apex of the deformity; dysplastic pedicles; widened spinal canal and foramen. 5

Regarding the surgical treatment of these spinal deformities in scoliosis secondary to NF1, when a combined arthrodesis (anterior and posterior) is performed with the use of an autologous graft taken from the resected ribs, the deformity can be corrected with stable instrumentation and a larger surgical area, in order to reduce the effects of continuous bone erosion in dystrophic cases. 5 6 In Marfan syndrome, the curves tend to be rigid and rapidly progressing, which indicates the need for surgical intervention. 7 8

Due to the scarce literature on the evaluation of pulmonary function in long-term follow-up after surgery for syndromic scoliosis, the present study aims to compare the long-term effect (> 10 years) of combined spinal fusion on pulmonary function in patients with scoliosis secondary to Marfan syndrome versus NF1.

## Materials and Methods

This was a case series study, conducted at a national referral center for spinal surgery, with the evaluation of the pulmonary function of 168 patients with scoliosis, operated from March 1997 to December 2009. The inclusion criteria were patients of both sexes with scoliosis secondary to Marfan syndrome and NF1 who underwent combined arthrodesis, with hooks and pedicle screws or only pedicle screws implants, without thoracoplasty and/or halo traction.

The exclusion criteria were patients with heart disease, pulmonary illnesses, infection, cognitive changes that influenced the understanding of the tests, and inability to perform the proposed evaluation. Eligible patients underwent an assessment during the last follow-up period with radiography and spirometry tests and were allocated into two groups for comparison: NF1 versus Marfan syndrome.

Data were obtained from medical records and follow-up visits, after approval by the institutional research ethics committee and signing of the consent form by the patients. During the follow-up, pulmonary function tests (PFTs) and radiographs (in anteroposterior and lateral) were performed, which were reviewed by two independent physicians not involved in the study, using the same terminal vertebrae used in the calculation of the preoperative Cobb angle. Data were collected of the following variables: sex; age (at diagnosis, at surgery, and at last follow-up); corrected height by wingspan; body mass index (BMI); duration of surgery (minutes); estimated blood loss (cc); follow-up (years); pulmonary and implant-related complications; pre- and postoperative Cobb angle of the main thoracic curve and thoracic kyphosis (T5 to T12); number of fused levels; predicted absolute and percentages values of forced vital capacity (FVC) and forced expiratory volume in 1 second (FEV1).

Pulmonary function tests were performed with the patient in the sitting position, performing the forced maximal expiration maneuvers. None of the patients reported being smokers. At least three acceptable curves and two reproducible curves were obtained, and the following parameters were recorded and classified according to the American Thoracic (ATS) and European Respiratory (ERS) societies' 2005 guidelines. 9

The diagnoses of Marfan syndrome cases followed the Ghent criteria, 10 which were updated in 2010. 11 However, in our study, the criteria in force at the time were used. The diagnosis of NF1 is based on clinical criteria, requiring two or more criteria. Dystrophic scoliosis in these patients presents with a single rigid thoracic curve (< 30% of flexibility), short and sharp; rib penciling; wedging of one or more vertebral bodies; masses in paraspinal or intraspinal tissues; marked rotation at the apex of the deformity; dysplastic pedicles; and widened spinal canal and foramen. 5 12

The choice of the combined approach was based on radiographic criteria (rigid curves, flexibility < 30%, and/or severe curves > 80°). After interbody anterior fusion the patient remained hospitalized in the intensive care unit and/or ward to undergo posterior fusion. In the posterior approach, posterior column osteotomies (PCOs) were performed at three to four levels. In all surgeries, the wake-up test was performed, because, during this period, the intraoperative neurophysiological evaluation with somatosensory and motor evoked potentials was not available in the institution.

### Statistical Analysis

Quantitative data were expressed as mean and standard deviation, submitted to the Kolmogorov-Smirnov normality test and compared between groups using Student's *t* test, Analysis of Variance (ANOVA)/Bonferroni (parametric data), and Mann-Whitney or Kruskal-Wallis/Dunn tests (nonparametric data). The intragroup analysis was performed using the paired t test (parametric data) or Wilcoxon test (nonparametric data). Categorical data were expressed as absolute and percentage frequencies and associated using Fisher's exact or Pearson's chi-square tests. All analyses were performed in the IBM SPSS Statistics for Windows (IBM Corp.), version 20.0, adopting a 95%CI.

## Results

From an initial population of 168 patients (221 spirometry), after undergoing the inclusion and exclusion criteria, a sample of 9 eligible patients was reached and evaluated with postoperative spirometry.

When analyzing the preoperative values of clinical and radiographic variables, as well as the pulmonary function test of patients with syndromic scoliosis with and without follow-up, found no significant differences between them ( Table 1 ).

**Table 1:** Comparison between preoperative clinical and radiographic variables of patients with versus without follow-up

	Follow-up		
	Yes	No	*p* -value
Mean age at the last PFT before surgery (years)	16.2 ± 3.9	18.6 ± 9.9	0.50 ^a^
Sex (F/M)	5/4	7/4	1.00 ^b^
Mean corrected height by wingspan (cm)	142.3 ± 56.3	151.7 ± 17.7	0.60 ^a^
Neurofibromatosis type 1/Marfan syndrome	5/4	8/3	1.00 ^b^
Mean Body Mass Index (kg/m ^2^ )	16.0 ± 1.8	16.3 ± 2.5	0.74 ^a^
Mean FVC (L)	1.5 ± 0.7	1.8 ± 1.2	0.39 ^a^
%	41.4 ± 12.9	57.1 ± 25.7	0.11 ^a^
Mean FEV1 (L)	1.2 ± 0.6	1.5 ± 1.0	0.42 ^a^
%	39.7 ± 14.9	54.0 ± 25.4	0.15 ^a^
Mean Main thoracic Cobb angle (°)	71.8 ± 13.3	92.2 ± 21.5	0.06 ^a^

**Abbreviations:** FVC, forced vital capacity; FEV1, forced expiratory volume in 1 second; PFT, pulmonary function test.

**Notes:**^a^ Student's *t-* test; ^b^ Fisher's exact test; * *p* < 0.05.

Furthermore, it was found that there was no significant difference between the groups in relation to the variables: age at surgery and at last follow-up, sex, preoperative corrected height by wingspan, preoperative BMI, fused levels, estimated blood loss, and follow-up time. This value was higher in patients with Marfan syndrome, approximately 16 years, but it was greater than 10 years in both groups ( Table 2 ).

**Table 2:** Comparison between clinical and radiographic parameters according to the etiology of syndromic scoliosis

	Marfan syndrome	NF1	*p* -value
**Mean age at surgery (years)**	15.2 ± 4.4	17.5 ± 3.4	0.42 ^a^
**Mean age at last follow-up (years)**	32.7 ± 8.3	31.4 ± 4.7	0.78 ^a^
**Sex: n (%)**			0.52 ^b^
Male	2 (40.0%)	3 (75.0%)	
Female	3 (60.0%)	1 (25.0%)	
**Mean preoperative corrected height by wingspan (cm)**	121.3 ± 69.5	168.5 ± 19.3	0.23 ^a^
**Mean preoperative Body Mass Index**	16.5 ± 2.0	15.4 ± 1.7	0.43 ^a^
**Mean fused levels**	11.8 ± 1.6	11.7 ± 1.7	0.97 ^a^
**Mean estimated blood loss (mL)**	2,624.2 ± 2,081.9	2,255.5 ± 723.5	0.75 ^a^
**Mean operative time (minutes)**	577.0 ± 178.1	598.7 ± 167.4	0.86 ^a^
**Preoperative FVC: n (%)**			
≤ 50%	3 (60.0%)	4 (100,0%)	0.44 ^b^
50–65%	2 (40.0%)	0 (0,0%)	
65–80%	0 (0.0%)	0 (0,0%)	
80–100%	0 (0.0%)	0 (0,0%)	
**Preoperative FEV1: n (%)**			
< 35%	1 (20.0%)	2 (50.0%)	0.52 ^b^
35–49%	2 (40.0%)	2 (50.0%)	
50–59%	1 (20.0%)	0 (0.0%)	
60–69%	1 (20.0%)	0 (0.0%)	
>70%	1 (20.0%)	0 (0.0%)	
**Mean follow-up (years)**	15.9 ± 4.5	12.4 ± 1.9 PM	0.20 ^a^

**Abbreviations:** FVC, forced vital capacity; FEV1, forced expiratory volume in 1 second; NF1, neurofibromatosis type 1.

**Notes:**^a^ Student's *t-* test; ^b^ Fisher's exact test; * *p* < 0.05.

Regarding the parameters of preoperative pulmonary function between the groups, it was found that half of the patients with Marfan syndrome had severe pulmonary involvement, while all those with neurofibromatosis had severe pulmonary involvement. It is worth noting that there were no differences between the groups in relation to these variables ( Table 2 ).

When comparing the preoperative and postoperative radiographic values between the groups, a significant reduction ( *p* < 0.05) of the main thoracic curve after surgery in the magnitude of the Cobb angle was observed in patients with Marfan syndrome. However, there was no difference in this variable between the groups, as well as in the preoperative and postoperative values of thoracic kyphosis ( Table 3 ).

**Table 3:** Involvement of pulmonary and radiographic parameters in patients with scoliosis secondary to Marfan syndrome and dystrophic NF1

	Marfan syndrome	Dystrophic NF1	*p* -value
**Mean FVC (L)**			
Preoperative	3.2 ± 1.2	3.9 ± 0.9	0.37 ^a^
Postoperative	2.3 ± 0.7	2.3 ± 1.7	0.96 ^a^
***p*** **-value**	0.29 ^b^	0.09 ^b^	
Post–Preoperative	-0.9 ± 1.7	-1.6 ± 1.26	0.55 ^a^
**Mean FEV1 (L)**			
Preoperative	1.3 ± 0.9	1.2 ± 0.3	0.87 ^a^
Postoperative	1.9 ± 0.7	1.9 ± 1.4	0.98 ^a^
***p*** **-value**	0.23 ^b^	0.36 ^b^	
Post–Preoperative	0.7 ± 1.1	0.7 ± 1.4	0.95 ^a^
**Mean FVC (%)**			
Preoperative	44.9 ± 16.6	37.0 ± 5.3	0.40 ^a^
Postoperative	39.6 ± 23.4	35.0 ± 12.2	0.73 ^a^
***p*** **-value**	0.67 ^b^	0.71 ^b^	
Post–Preoperative	-5.3 ± 25.6	-2.0 ± 9.9	0.82 ^a^
**Mean FEV1 (%)**			
Preoperative	43.4 ± 19.4	35.0 ± 6.7	0.44 ^a^
Postoperative	53.2 ± 14.3	34.7 ± 11.3	0.07 ^a^
***p*** **-value**	0.20 ^b^	0.95 ^b^	
Post–Preoperative	9.8 ± 14.5	-0.3 ± 8.4	0.26 ^a^
**Mean Cobb angle of main thoracic curve (°)**			
Preoperative	74.0 ± 16.3	69.0 ± 10.0	0.61 ^a^
Postoperative	46.2 ± 18.0	68.2 ± 21.2	0.13 ^a^
***p*** **-value**	** 0.04 ^b^**	0.96 ^b^	
Post–Preoperative	-27.8 ± 20.5	-0.7 ± 26.9	0.13 ^a^
**Mean thoracic kyphosis (T5–T12) (°)**			
Preoperative	30.0 ± 14.1	–	–
Postoperative	56.2 ± 28.2	–	–
***p*** **-value**	0.18 ^b^	–	–
Post–preoperative	41.0 ± 17.0	–	–

**Abbreviations:** FVC, forced vital capacity; FEV1, forced expiratory volume in 1 second; NF1, neurofibromatosis type 1.

**Notes:**^a^ Student's *t* test; ^b^ Paired *t* test; * *p* < 0.05.

When analyzing the absolute and predicted percentages values of preoperative and postoperative FEV1 and FVC, there were no significant differences within the same group, or between them, during the last follow-up ( Table 3 ).

When evaluating the odds ratio (OR) between the etiology of syndromic scoliosis versus pulmonary complications in patients undergoing dual-approach surgery, there was no greater chance of pulmonary complications of one syndrome compared to the other ( Table 4 ).

**Table 4:** Analysis of the odds ratio between the presence of postoperative pulmonary complications versus the etiology of scoliosis

	Pulmonary complications		*p* -value	Odds ratio (95%CI)
	Yes	No		
**Etiology of the scoliosis**			0.20	
Marfan syndrome	1 (25.0%)	4 (80.0%)		12.0 (0.5–28.1)
Dystrophic neurofibromatosis type 1	3 (75.0%)	1 (20.0%)		

**Notes:** Fisher's exact test; * *p* < 0.05.

In the three patients with NF1 who underwent thoracotomy followed by posterior arthrodesis, the complications were: atelectasis, pneumothorax, hemothorax, and pachypleus in one patient; pneumonia and respiratory failure in another; and pneumonia in the last one. In the Marfan syndrome case, the patient underwent a similar surgical approach, and the pulmonary complication was atelectasis.

## Discussion

In the present study, patients with a defined syndromic diagnosis were evaluated, who were divided into two groups: Marfan and dystrophic NF1, with a follow-up time of more than 10 years. In the first, the patients had rigid curves (< 30% of flexibility) and in the second, in addition to stiffness, the presence of dystrophic/dysplastic characteristics. This influenced the choice of the combined approach.

Surgical cases for patients with scoliosis due to Marfan syndrome usually involve more fused levels. In these cases, the anterior release, by removing the intervertebral discs around the apex of the deformity, promotes greater flexibility of the curve, reducing the shear forces between the interface of the implants and the fixation bone structures. 2

In our study, the mean number of fused levels between the two groups (Marfan syndrome and NF1) did not differ, being 11.80 ± 1.6 and 11.75 ± 1.7 respectively, higher than that used in cases of selective arthrodesis, where the maximum level is around 8 to 10 for thoracic curves.

Patients with scoliosis lower than 55° may already have a decline in forced vital capacity. It is worth noting that the more cephalic the apex of the deformity and the lower the patient's kyphosis, the greater the impairment of pulmonary function. 4

In the present series, the patients had a mean thoracic kyphosis of 30 ± 14.1° and the Cobb angle of the main thoracic curve of 74 ± 16.3° in the preoperative period. Regarding preoperative pulmonary involvement, it was observed that approximately half of the patients had severe impairment with percentages of FEV1 lower than 50% and the majority had a FVC ≤ 50%.

A study that evaluated the preoperative parameters of pulmonary function in patients with scoliosis secondary to Marfan syndrome found no correlation between the preoperative values of thoracic kyphosis (Cobb T5–T12) and curve magnitude (Cobb of the thoracic curve in the coronal plane) with FVC and FEV1 values. In this study, the mean percentages of FVC and FEV1 were of 70.12 ± 22.40 and 67.07 ± 20.213% respectively. 3

In our series, in the group of patients with Marfan syndrome, there was a significant reduction in the magnitude of the curve (74.0→46.2°) after the surgical procedure. Regarding pulmonary function assessment, there was no significant variation in the predicted percentages of FVC (-5.28 ± 25.65%) and FEV1 (9.82 ± 14.46%) between the preoperative period and the last follow-up.

In a cohort of patients with scoliosis secondary to Marfan syndrome who underwent surgery by dual approach, surgical complications were found to be screw loosening in two patients, stem break in one, and chylothorax with hemothorax in the patient who underwent thoracoscopy. 2

When evaluating the complications in patients with Marfan syndrome, we observed one case of atelectasis that was managed with antibiotics and respiratory physiotherapy, with resolution of the condition. No implant-related complications were observed.

The other group studied refers to patients with scoliosis secondary to NF1 with a dystrophic pattern, characterized by a single rigid, short, and sharp thoracic curve; rib penciling; wedging of one or more vertebral bodies; masses in paraspinal or intraspinal tissues; marked rotation at the apex of the deformity; dysplastic pedicles; and/or widened spinal canal and foramen. According to certain authors, in the presence of three or more of these characteristics, the mean chance of progression of the deformity is 85%, with a risk of impaired pulmonary function in these patients. 5

In our study, patients diagnosed with NF1 had scoliosis with a rigid and short thoracic curve (preoperative Cobb: 69 ± 10°), associated with dysplastic pedicles and changes in the costal arches (rib penciling). This was associated with severe preoperative pulmonary involvement, with FVC and FEV1 lower than 50%.

Certain authors suggest that, in dystrophic cases, surgery should be performed as soon as possible, due to the rapid progression of the curve. The fact that arthrodesis is performed in younger patients would not necessarily result in a loss in their final height, as the number of fused levels would be lower. 6

In our study, patients were operated, on average, after the growth spurt and near the end of adolescence (∼ 17 years) with a preoperative corrected height of 168.50 ± 19.3 cm, with an average of about 12 levels fused after surgery.

A study that analyzed the results of surgery combined with thoracotomy in patients with early-onset dystrophic scoliosis (< 10-years-old) showed a reduction in the magnitude of the curve (71.2→23.5°) and changes in the preoperative and postoperative values of FVC (1.4→2.3 L) and FVC (75.0→74%). 5

The present authors did not find a significant improvement in the Cobb angle of the main thoracic curve after surgery, as well as in the variation (postoperative – preoperative) of pulmonary function parameters: FVC (-1.57 ± 1.26L), FEV1 (0.76 ± 1.40L), and percentages (FVC: −2.00 ± 9.94%; and FEV1: −0.28 ± 8.41%).

When evaluating the results of different studies on patients with scoliosis secondary to NF1 with a dystrophic pattern, Jia et al. showed that there was a significant reduction in the Cobb angle of the main thoracic curve in patients who underwent combined arthrodesis and posterior arthrodesis alone, with no difference between the two groups. According to them, both approaches have similar efficacy, stability, and safety in the postoperative follow-up of these patients. 13

Regarding complications, after anterior and posterior approach to these patients, certain authors reported that about 28.5% of them evolved with complications, such as: malpositioning and loosening of the implants, dural fistula, pseudarthrosis, atelectasis, pressure ulcer, superficial infection, transient paraplegia and paraparesis, crankshaft phenomenon, among others. They point out that the proportion of instrumentation failure or pseudarthrosis was 8.3%. 13

Our study found that three patients with NF1 who underwent thoracotomy followed by posterior arthrodesis were complicated with: atelectasis, pneumothorax, hemothorax, and pachippleuris in one patient treated with chest drainage and pulmonary physiotherapy; pneumonia and respiratory failure in another, who underwent orotracheal intubation and intravenous antibiotic therapy; and pneumonia in the last patient. All of them progressed well clinically. It should be noted that none of the patients developed complications related to the implants.

When comparing the two groups of patients in the present study, it was observed that, although patients with NF1 had lower values (worse involvement) of the preoperative pulmonary function parameters and in the last follow-up compared to the group with Marfan syndrome, this difference was not significant. Additionally, there was no greater chance of pulmonary complications of one etiology than the other, using the same surgical approach.

As strengths of our research, we can mention this is one of the few studies that presents pre- and postoperative pulmonary function parameters in syndromic scoliosis patients with a long follow-up (greater than 10 years). However, caution must be taken when interpreting the results and in their external validation, due to the inherent limitations of this research.

The limitations can be attributed to the study's retrospective, single center nature; having a small sample; and not having evaluated the effect of only posterior spinal fusion, skeletal traction, vertebral osteotomies, and thoracoplasty on pulmonary function.

## Conclusion

When evaluating the effect of combined spinal fusion (anterior and posterior) on pulmonary function in patients with syndromic scoliosis (Marfan syndrome and NF1) in long-term follow-up (> 10 years), it was found that there was no difference in the absolute and predicted percentages of FVC and FEV1 between the groups in the last follow-up. In addition, pulmonary function values did not show significant changes in the last postoperative evaluation compared to the preoperative period.
